# Degradation of PsbO by the Deg Protease HhoA Is Thioredoxin Dependent

**DOI:** 10.1371/journal.pone.0045713

**Published:** 2012-09-19

**Authors:** Irma N. Roberts, Xuan Tam Lam, Helder Miranda, Thomas Kieselbach, Christiane Funk

**Affiliations:** Department of Chemistry and Umeå Plant Science Centre, Umeå University, Umeå, Sweden; Friedrich-Alexander-University Erlangen-Nurenberg, Germany

## Abstract

The widely distributed members of the Deg/HtrA protease family play an important role in the proteolysis of misfolded and damaged proteins. Here we show that the Deg protease rHhoA is able to degrade PsbO, the extrinsic protein of the Photosystem II (PSII) oxygen-evolving complex in *Synechocystis* sp. PCC 6803 and in spinach. PsbO is known to be stable in its oxidized form, but after reduction by thioredoxin it became a substrate for recombinant HhoA (rHhoA). rHhoA cleaved reduced eukaryotic (specifically, spinach) PsbO at defined sites and created distinct PsbO fragments that were not further degraded. As for the corresponding prokaryotic substrate (reduced PsbO of *Synechocystis* sp. PCC 6803), no PsbO fragments were observed. Assembly to PSII protected PsbO from degradation. For *Synechocystis* sp. PCC 6803, our results show that HhoA, HhoB, and HtrA are localized in the periplasma and/or at the thylakoid membrane. In agreement with the idea that PsbO could be a physiological substrate for Deg proteases, part of the cellular fraction of the three Deg proteases of *Synechocystis* sp. PCC 6803 (HhoA, HhoB, and HtrA) was detected in the PSII-enriched membrane fraction.

## Introduction

Cells have evolved an extensive system of molecular chaperones, folding catalysts, and proteases; that control protein quality and prevent protein damage. Biochemical and molecular biological studies have successfully identified many plastidic protease families, most of which are homologs of defined bacterial proteases.

Deg/HtrA proteases were initially identified because of their essential role in the degradation of abnormal periplasmic proteins and because they are crucial for the survival of *E. coli* at high temperatures [1], [2]. They have since been found in nearly all organisms; including Archae, bacteria and eukaryotes. Deg proteases are ATP-independent serine endopeptidases, containing a trypsin/chymotrypsin-like protease domain, followed by zero to three PDZ or PDZ-like domains [3], [4]–PDZ domains being those that mediate complex assembly, substrate binding, and/or regulation of proteolytic activity [5]–[7]. In *Arabidopsis thaliana*, 16 genes coding for Deg-like proteases have been identified and at least seven gene products have been predicted to be located in chloroplasts [8]–[10]. Based on biochemical data, five Deg proteases have been shown to be localized in the chloroplast: Deg1, Deg5, and Deg8 are located in the thylakoid lumen [11], [12], and Deg2 [13] and Deg7 [14] are peripherally associated with the stromal side of the thylakoid membrane. Degradation of the Photosystem II (PSII) reaction-center protein D1 under photoinhibition has been linked to the lumen-located Deg1 [15], and to Deg5 and Deg8 [16]. Additionally, Deg1 seems to assist the assembly of PSII via interaction with the PSII reaction-center D2 protein [17].

It has been shown *in vitro* that recombinant Deg1 of Arabidopsis is able to degrade *in vitro*–translated plastocyanin and PsbO [18]. So far, no details of the molecular mechanism of Deg function are known, but recently it has been suggested that some Deg proteases might be redox regulated. In one study it was found that the proteolysis of casein by recombinant Deg1 and Deg2 of Arabidopsis was dependent on the redox potential of the surrounding medium; and while the activity of Deg1 was maximal under reducing conditions, the opposite was true for Deg2 [19]. Deg1 and Deg5 have been identified as potential thioredoxin targets from their ability to form mixed disulfides with Trx A (thioredoxin A) of the unicellular cyanobacterium *Synechocystis* sp. PCC 6803 (hereafter: *Synechocystis* 6803) [20].

In *Synechocystis* 6803, three Deg/HtrA proteases have been identified. Owing to their relationship to *E. coli* they have been named DegP/HtrA (*slr1204*), DegQ/HhoA (*sll1679*), and DegS/HhoB (*sll1427*) [21]. However, they are more closely related to each other than to the *E. coli* Deg proteases with the same name [8], [9]; so they are probably not orthologs of the *E. coli* Deg proteases. HhoA, HtrA, and HhoB have very high homology with the lumen-located plant Deg proteases Deg1, Deg5, and Deg8 [8].

Single-deletion mutants of HtrA [22] and HhoA [23] have been found to be more sensitive than wild type, towards light and heat stress, respectively. However, these results were controversial [24], [25]. While a triple Deg-deletion mutant displayed a significant phenotype towards light- and heat-stress and phototaxis, none of the double mutants did. Therefore it was concluded that the Deg proteases of *Synechocystis* 6803 have at least partially overlapping functions [25].

PSII, which catalyzes light-dependent water oxidation and plastoquinone reduction in plants and cyanobacteria, consists of more than 30 proteins located in the thylakoid membrane. The catalytic reaction center as well as the chlorophyll (Chl)-binding proteins are membrane integral, but they are stabilized by several extrinsic proteins bound to the lumenal surface of PSII [26], [27], called the oxygen-evolving complex (OEC). While the extrinsic protein PsbO is present in all organisms that perform oxygenic photosynthesis [28], [29], the higher-plant PsbP and PsbQ differ from the corresponding proteins PsbU and PsbV present in the cyanobacterial water-oxidizing complex.

Although none of its amino acid residues are likely ligands to the Mn_4_Ca cluster, PsbO has been found to play an important role in the stabilization of the oxygen-evolving complex; and after removal of PsbO the manganese ions are released. However, oxygen-evolving activity is maintained in the presence of high concentrations of Cl^−^ and Ca^2+^ [30]. Besides being important for the stabilization of the manganese cluster, PsbO has also been shown to be involved in many other aspects of PSII structure and function [31]–[34].

PsbO has not yet been crystallized owing to its natively unfolded nature [35]. The only experimental three-dimensional structure of PsbO was derived from cyanobacterial PsbO bound to PSII [26], [36] and has served as a template for the construction of homologous models for plant PsbO [29]. It has been found that pH values of 5.7 and 7.2–which are typical for the light and dark conditions in the thylakoid lumen–change the conformation of PsbO [37]. PsbO has two conserved cysteine residues, which correspond to Cys19 and Cys44 in the cyanobacterium *T. elongatus* [28] and to Cys28 and Cys51 in spinach. These cysteines form a disulfide bridge between the N-terminal loop and the β1 strand [38]. The role of this disulfide bond is controversial; it has been observed to be involved in accumulation of PsbO at the thylakoid membrane [39], and in its rebinding to PSII [40]. However, after deletion of the disulfide bond, a PsbO Cys_28_Ala/Cys_51_Ala double mutant was still able to assemble PsbO to PSII and to restore oxygen evolution up to 40% of the control level [41], [42].

Unlike unassembled intrinsic subunits of PSII, which are rapidly degraded; a pool of free, assembly-competent, extrinsic OEC proteins has been shown to exist in the thylakoid lumen [43], [44]. The availability of soluble OEC proteins is thought to be important for the rapid reassembly of functional oxygen-evolving PSII complexes during the PSII repair cycle. PsbO has been shown to have a long lifetime even in its free form [44], but it can be oxidatively damaged under light stress [45]. Recent *in vitro* studies have shown that the PsbO proteins of Arabidopsis and spinach are targets of thioredoxin [20], [46]–[48]. Notably, the disulfide bridge of PsbO was not only reduced by thioredoxin, but the redox state of the disulfide was integral to the degradation of PsbO1 and PsbO2 of Arabidopsis [20].

The protease activities responsible for the redox-dependent proteolysis have not yet been identified. Here we demonstrate that recombinant HhoA of the cyanobacterium *Synechocystis* 6803 is able to degrade PsbO from spinach in a redox-dependent manner, and we present the corresponding cleavage sites. In agreement with earlier observations in Arabidopsis [20], we observed that PsbO degradation was induced after reduction of the disulfide bond in both spinach and *Synechocystis* 6803. We provide evidence that the redox-dependent degradation of PsbO in cyanobacterial thylakoid membranes is performed by Deg proteases. Finally, we demonstrate the subcellular localization of the three Deg proteases to be at the thylakoid membrane and/or in the periplasmic space.

## Results

### rHhoA is able to degrade cyanobacterial PsbO in a redox-dependent manner

It was recently shown by means of proteomics that the redox state of the cysteine thiols is important for the stability of both PsbO1 and PsbO2 in Arabidopsis [20]. The proteases known to be located in the plant chloroplast lumen are the D1-processing proteases and the Deg proteases Deg1, Deg5, and Deg8. Deg1 is the most abundant protease in the soluble lumen content and it is therefore a reasonable assumption that it would be involved in the redox-dependent degradation of PsbO. However, Deg1 of Arabidopsis was recently reported to be redox regulated itself [19]. In addition, Deg1 and Deg5 of Arabidopsis have been shown to form mixed disulfides with TrxA of *Synechocystis* 6803 [20], suggesting that the lumenal Deg proteases of green plants might be redox regulated. To unambiguously investigate the redox-dependent degradation of the substrate PsbO–and not of the protease itself–we designed *in vitro* degradation experiments using cyanobacterial recombinant Deg proteases. All three *Synechocystis* 6803 Deg proteases are highly homologous to the three lumen-located Deg proteases of Arabidopsis, but not to the other Deg proteases of this plant [8]. While HhoA and HhoB are free of cysteines, HtrA contains one N-terminal cysteine, which, however, is not conserved among HtrA of other organisms, suggesting that HtrA is not regulated via redox-active thiols. In addition, no Deg protease was detected among the thioredoxin targets of *Synechocystis* 6803 [49]. Therefore, the activities of HtrA and the other Deg proteases of *Synechocystis* 6803 are not controlled by thioredoxin.

**Figure 1:**
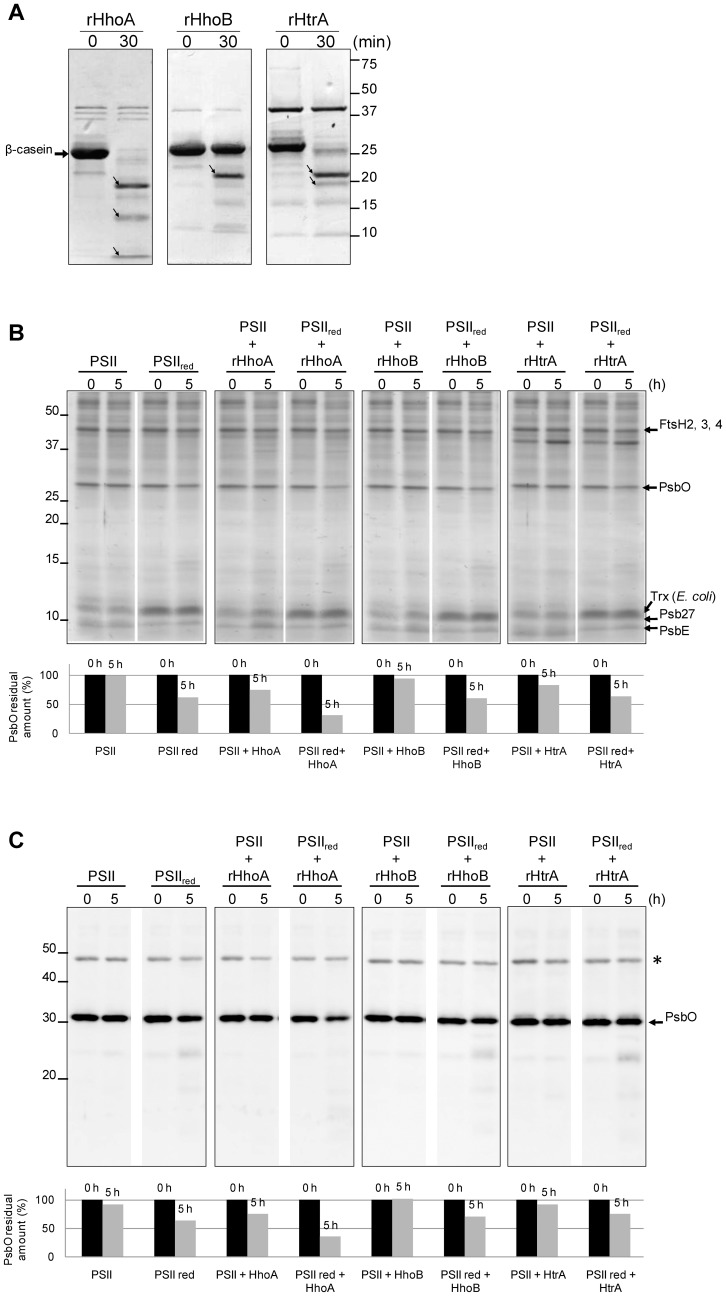
Cyanobacterial PsbO is degraded by rHhoA in a redox-dependent manner. (A) Proteolytic activity of recombinant *Synechocystis* 6803 Deg/HtrA proteases against naturally unfolded β-casein. Small arrows indicate β-casein degradation fragments. (B) A PSII-enriched fraction was isolated from *Synechocystis* 6803 using the HT3 mutant with His-tagged CP47. The PSII-enriched fraction was incubated for 5 h in the absence (PSII) or the presence (PSII_red_) of the thioredoxin system, either with no protease (panel 1) or with rHhoA (panel 2), rHhoB (panel 3), or rHtrA (panel 4). The proteins (15 µg) were separated by SDS-PAGE and analyzed with CBB staining. Identity of the named bands was confirmed using mass spectrometry. (C) After SDS-PAGE proteins were transferred to PVDF membranes and immunostained using an antibody directed against PsbO. Bars below the gels and blots show the integrated density values of the corresponding bands as quantified by Image J software. The asterisk marks an nonspecific cross-reacting band.

Activity of the three purified recombinant Deg proteases of *Synechocystis* 6803–rHhoA, rHhoB, and rHtrA–was confirmed using β-casein as a substrate (Figure 1A). All three recombinant proteases exhibited proteolytic activity, as had previously been demonstrated [50]. To investigate if redox-dependent degradation of PsbO could be observed in *Synechocystis*, we performed an *in vitro* proteolytic assay using a PSII-enriched fraction isolated from the HT3 strain as the source of PsbO. The *Synechocystis* HT3 strain expresses a His-tagged CP47 protein; which allows purification of a highly enriched PSII fraction by nickel-affinity chromatography [51], [52]. In agreement with previous reports [52], mass spectrometric analysis of major bands in this fraction allowed identification of several PSII subunits (Figure 1B). The isolated PSII-enriched fraction was incubated with each of the three recombinant Deg proteases for 5 h under reduced conditions. The reduced conditions were conferred using the complete *E. coli* thioredoxin system, consisting of thioredoxin (Trx), thioredoxin reductase (TrxR), and β-NADPH. Figure 1B shows the CBB R-250 stained gels and Figure 1C shows the immunoblots using an anti-PsbO antibody. As seen in Figures 1B and C, PsbO in the PSII complex was moderately degraded in the presence of reduced thioredoxin (PSII_red_), while no degradation was detected when reduced thioredoxin was absent (panel 1). Quantification analysis of the signals shows that 63% of PsbO initial amount remained after 5 h (PSII_red_). We believe that this degradation can be attributed to low amounts of *Synechocystis* proteases co-isolating with the PSII-containing membrane fractions. Nonetheless, addition of recombinant HhoA clearly increased the degradation of PsbO in the presence of thioredoxin, as can be observed both in the CBB-stained gels (Figure 1B) and in the immunoblots (Figure 1C) where about 30 and 35% of the initial amount of PsbO remained after 5 h, respectively. On the other hand, addition of rHhoB or rHtrA did not enhance the degradation of reduced PsbO bound to PSII. It is noteworthy that no additional PsbO fragments were observed in the CBB-stained gels or in the immunoblots (Figure 1C), therefore either the antibody did not recognize the degradation products, or PsbO degradation in this experiment was complete.

### Subcellular location of the Deg proteases of *Synechocystis* 6803

**Figure 2:**
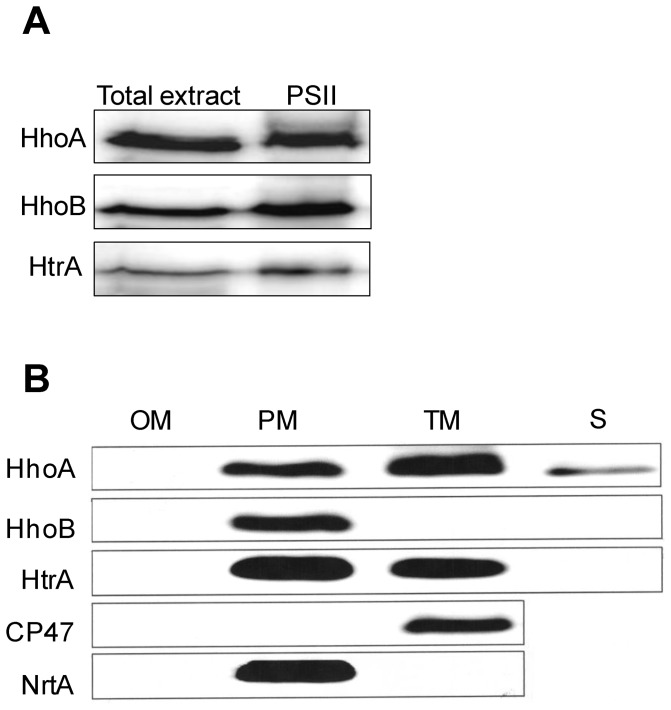
Subcellular localization of the *Synechocystis* Deg proteases. (A) Total wild-type cell extract and a PSII-enriched fraction isolated from the HT3 strain of *Synechocystis* 6803 were analyzed by SDS-PAGE, and by immunostaining using antibodies directed against recombinant HhoA, HhoB, or HtrA. Of the total cell extract, samples corresponding to 0.1 µg of chlorophyll were loaded for immunostaining with anti-HhoA and samples corresponding to 0.5 µg were loaded for anti-HhoB and anti-HtrA. Of the PSII-enriched fraction, samples corresponding to 5 µg of chlorophyll were loaded in each lane. (B) Outer membrane (OM), plasma membrane (PM), thylakoid membrane (TM), and soluble protein fraction (S) were isolated from *Synechocystis* 6803 by gradient centrifugation and two-phase partitioning, separated by SDS-PAGE, and immunostained using antibodies directed against recombinant HhoA, HhoB, or HtrA. Purity of the membranes was determined by using antibodies directed against the PSII protein CP47 and against NrtA, a component of the nitrate transporter; 15 µg of protein was loaded in each lane.

To investigate if the internal degradation of reduced PsbO observed in the PSII-enriched fraction (Figure 1B and 1C, panel 1) could be attributed to native cyanobacterial Deg proteases, PSII-enriched membranes were isolated from the HT3 strain. After SDS-PAGE, the proteins were immunostained with antibodies directed against HhoA, HhoB, or HtrA. As shown in Figure 2A, all three Deg proteases were detected in the PSII-enriched fraction. However, it is important to note that the PSII-enriched fraction had to be concentrated 10–50 times to achieve immune signals of the same intensity as for the total cell extract. These data suggest that even though all three Deg proteases can be found in the membrane fraction in proximity to PSII, they are present in substoichiometric amounts.

Earlier proteomic studies have identified HtrA in the outer membrane of *Synechocystis* 6803 [53]; and HhoA has been detected in the periplasm, where it has been found both in a soluble form [54] and in a plasma membrane–bound form [55]; while the subcellular location of HhoB is still unknown. To further investigate the subcellular location of the Deg proteases within the membrane system of *Synechocystis* 6803, the different membrane fractions were isolated in a two-dimensional manner using density-gradient centrifugation and two-phase partitioning [56]. As shown in Figure 2B, all three Deg proteases were immuno-localized in the plasma membrane of *Synechocystis* 6803. Additionally, HhoA and to a lesser extent HtrA were detected in the thylakoid membrane. The purity of the membrane fractions was controlled immunologically by using antibodies directed against the PSII core protein CP47, which is known to be localized in the thylakoid membrane, and against NrtA–a component of the nitrate transporter–, which is localized in the plasma membrane.

### Native PsbO is degraded in a redox-dependent manner in the thylakoid lumen of spinach

To further study the redox-dependent degradation, a pure PsbO fraction was needed that was free of contaminating proteases (native Deg/HtrA, or other proteases within the cell). To our knowledge, no method has been reported that allows the purification of large quantities of PsbO from *Synechocystis* cells. In addition, an attempt to express recombinant *Synechocystis* PsbO in two different *E. coli* strains resulted in the production of insoluble aggregates and a low fraction of apparently misfolded protein, highly prone to precipitation (data not shown). Analysis of the redox state of soluble recombinant PsbO showed that the protein is synthesized mostly in its reduced form, suggesting that *E. coli* is not able to correctly form the disulfide bridge (Figure S1). As a consequence, studies using purified PsbO from *Synechocystis* as substrate were not possible, and hence the usefulness of using PsbO purified from other sources was evaluated.

The amino acid sequence and structure of PsbO from prokaryotic and eukaryotic organisms are highly conserved [29]. The PsbO protein of *Synechocystis* has 43% sequence identity with its spinach and *Arabidopsis* counterparts (Figure S2), mostly within highly conserved regions [29]. Critical for similarity of protein structures is conservation of the amino acid residues that stabilize the hydrophobic core of a protein [57]. For PsbO from spinach, the molecular interactions of the individual amino acid residues that stabilize the protein core have been calculated [38]. PsbO from spinach has 107 amino acid residues that contribute with ten or more molecular interactions to the stability of the protein fold [38], and 56 of these amino acid residues (52%) are directly conserved in the sequence of PsbO from *Synechocystis*. In addition, both PsbO from spinach and from *Synechocystis* can without difficulty be aligned to the sequences of PsbO from *Thermosynechococcus*, for which experimental structures are available (PDB IDs: 1FE1, 1LX, and 1IZL). The sequence identity between these proteins is 35% or higher. This high degree of similarity of the sequences of cyanobacterial PsbO and spinach PsbO indicates that the folds of these proteins are most likely very similar and that spinach PsbO is a useful model for the proteolytic cleavage of cyanobacterial PsbO by Deg proteases.

For purification of PsbO, spinach has the advantage that it has a simpler arrangement of PSII that allows isolation of a homogeneous PsbO fraction. Multi-gene families coding for PsbO isoforms have been reported in Arabidopsis [11], [12], [58], pea [59], tomato [60], and tobacco [61]. Genomic sequencing of other plant species, such as rice and wheat, has also revealed multiple *PSBO* genes, coding for highly similar PsbO proteins. However, spinach has only one known PsbO gene product, and there is no evidence for the presence of other PsbO proteins in this plant [11].

**Figure 3:**
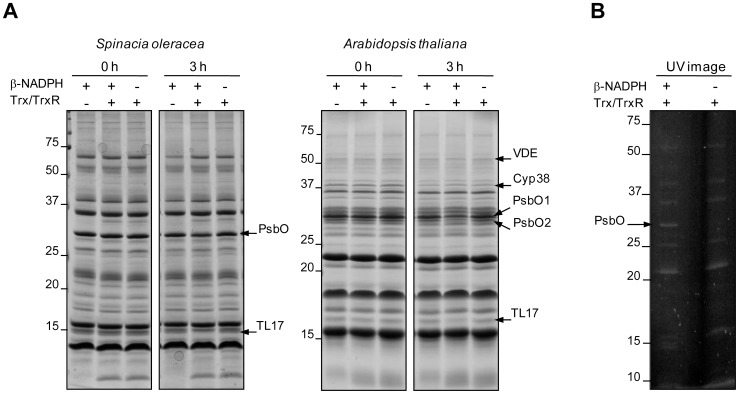
PsbO degradation in the thylakoid lumen of spinach and Arabidopsis is redox dependent. (A) Thylakoid lumen of spinach and Arabidopsis was isolated and incubated for 3 h in the presence of the complete thioredoxin system. Controls lacked either β-NADPH or thioredoxin (Trx) and thioredoxin-reductase (TrxR). After SDS-PAGE, the proteins (20 µg) were stained with CBB. Arrows indicate the position of the PsbO protein band in spinach, and the positions of PsbO1 and PsbO2 in Arabidopsis samples and of other prominent proteins identified by mass spectrometry. The remaining PsbO amount after 3 h of incubation was quantified using Image J software and was as it follows: 61, 46 and 41% in the presence of the complete thioredoxin system; 84, 81 and 98% in the controls lacking Trx and TrxR; and 82, 76 and 83% in the controls lacking β-NADPH for spinach PsbO, PsbO1 and PsbO2, respectively. (B) Thylakoid lumen of spinach was isolated and incubated with the complete thioredoxin system (Trx/TrxR/β-NADPH) or with thioredoxin and thioredoxin reductase (Trx/TrxR) as a control. Sulfhydryl groups of the lumenal proteins were labeled with mBBr. After SDS-PAGE, proteins were visualized with UV. Identity of the named bands was confirmed using mass spectrometry.

The extrinsic PsbO protein binds to the lumen side of PSII, and it is also present as soluble unassembled protein in the thylakoid lumen [44]. It is well known that the intrinsic proteolytic activity in the thylakoid lumen is low under non-reducing conditions, and most proteins of this compartment are not significantly degraded *in vitro* [62]. The recently reported observation that both PsbO subunits are degraded in Arabidopsis lumen samples in the presence of reduced thioredoxin [20] points to a potential participation of lumenal proteases in this process. To investigate if redox-dependent degradation of PsbO could also be observed in the thylakoid lumen of spinach, lumenal proteins were isolated from both spinach and Arabidopsis, and incubated in the presence of the complete thioredoxin system (Figure 3A). Control assays were performed using the thioredoxin system without the electron donor β-NADPH or vice versa. Even when some basal level of proteolysis was detected in the controls, PsbO degradation was clearly enhanced in the presence of reduced thioredoxin. However, redox-dependent degradation of PsbO was slower in the fraction of lumenal proteins from spinach than in that from Arabidopsis. Densitometric analysis of the corresponding bands revealed that about 61% of the initial PsbO amount remained in the spinach sample after 3 h of incubation in the presence of the complete thioredoxin system while for PsbO1 and PsbO2 from Arabidopsis 46 and 41% remained, respectively. As previously reported, redox-dependent (TL17) and -independent (Cyp38) protein degradation has been observed in the lumen of Arabidopsis [20] and redox-dependent degradation of TL17 is also clearly detectable in the lumenal fraction from spinach. The ability of reduced thioredoxin to reduce the disulfide of PsbO was confirmed using monobromobimane (mBBr), which allows specific fluorescence labeling of sulfhydryl groups (Figure 3B).

### rHhoA is able to degrade spinach PsbO in a redox-dependent manner

**Figure 4:**
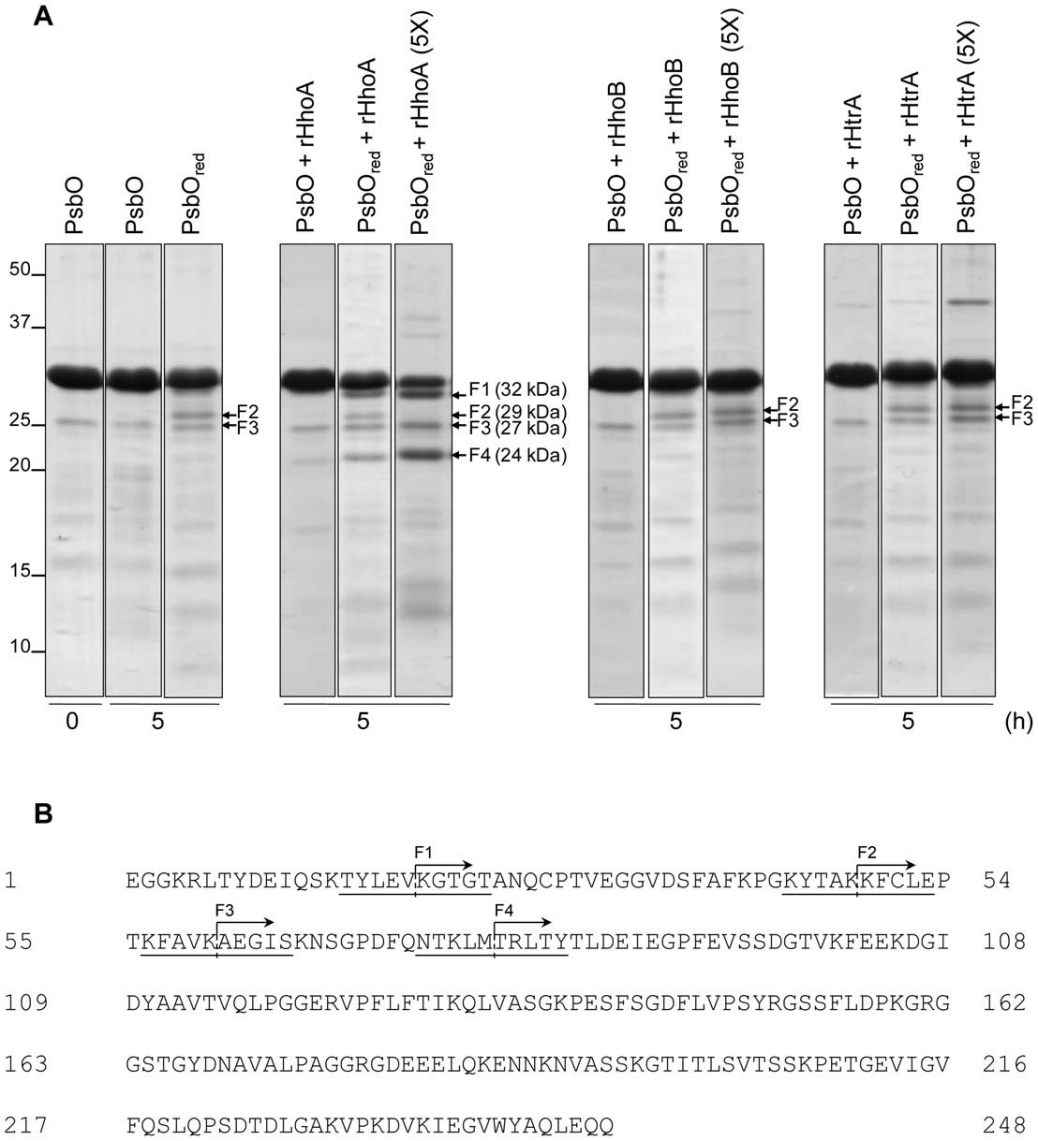
PsbO degradation in the presence of recombinant Deg proteases of *Synechocystis* sp. PCC 6803. (A) PsbO from spinach was isolated and incubated in the absence or the presence (PsbO_red_) of the thioredoxin system for 5 h either without addition of recombinant proteases (panel 1) or after addition of rHhoA (panel 2), rHhoB (panel 3), or rHtrA (panel 4) at two different enzyme-to-substrate ratios, 1∶50 and 1∶10 (5×). After SDS-PAGE, the proteins (9 µg) were stained with CBB. Arrows indicate the position of PsbO degradation fragments (F1 to F4). (B) The degradation fragments of reduced spinach PsbO produced by recombinant HhoA were transferred onto PVDF membrane, stained using CBB, and analyzed by Edman sequencing. The cutting sites are indicated by dashed lines in the mature PsbO sequence.

In order to test the susceptibility of spinach PsbO to *Synechocystis* Deg proteases, PsbO isolated from spinach leaves was incubated for 5 h at 37°C in the presence of the recombinant proteases rHhoA, rHhoB, and rHtrA (Figure 4A). No degradation was observed after 5 h in non-reducing conditions (panel 1, lanes 1 and 2); the weak bands visible in these lanes were not immunostained by the antibody directed against PsbO nor did mass spectrometric analysis identify any PsbO fragments (not shown). In the presence of the thioredoxin system (PsbO_red_) some residual degradation was observed that might be caused by low amounts of cross-contamination by spinach proteases (panel 1, lane 3). Longer incubation times were tested up to 10 h, and the time course degradation of PsbO was quantified from the CBB stained gels using the Image J software (Figure S3). Results showed that the fastest PsbO degradation was achieved in the presence of reduced thioredoxin and rHhoA in a ratio of 1∶10 enzyme to substrate (5×). In this case, about 38% of the initial PsbO remained after 5 h of incubation and less than 7% after 10 h. Concomitant with the decrease of the PsbO band at 33 kDa, an additional band with a molecular mass of 29 kDa was observed in the CBB-stained gel. Addition of recombinant HhoA to reduced PsbO using an enzyme to substrate ratio of 1∶50 resulted in further, strong degradation of the PsbO protein (panel 2, lane 2). Four degradation products were observed with apparent molecular masses of around 32 kDa (F1), 29 kDa (F2), 27 kDa (F3), and 24 kDa (F4). At higher rHhoA concentration (5×) the 24-kDa band accumulated, while the 29-kDa band disappeared (panel 2, lane 3). It is important to note that addition of recombinant HhoB or HtrA did not lead to any redox-dependent degradation of PsbO, apart from the residual degradation that was observed as background activity when no protease was added (panels 1, 3, and 4). This background activity was always around 20% of degradation (80% of initial PsbO remaining) even in the presence of recombinant HhoB and HtrA both at standard or high protease concentrations (5×) and after 10 h of incubation (Figure S3). Also addition of CaCl_2_ to the buffer to stimulate the activity of these proteases did not lead to higher degradation rates (not shown) [50].

Of the four fragments detected, F1 and F4 were specific bands, which only appeared in the presence of the thioredoxin system and rHhoA. F3 already was visible in unreduced PsbO preparations, but accumulated over time in the presence of the thioredoxin system and rHhoA (Figure 4A, panel 2, lane 3). In contrast, F2 was produced in the presence of the thioredoxin system without addition of recombinant proteases, and most likely it was the product of background protease activity. Interestingly fragment F3 was further degraded by rHhoA; its intensity in the CBB-stained gel was reduced over time and totally disappeared at higher rHhoA concentrations (Figure 4A, panel 2, lane 3). The proteolytic fragments of the redox-dependent PsbO degradation by recombinant HhoA were N-terminally sequenced using the Edman degradation method (Figure 4B). The cleavage sites in spinach PsbO were identified as Val19–Lys20 (F1), Lys48–Lys49 (F2), Lys60–Ala61 (F3), and Met78–Thr79 (F4) (Figure 4B). The rHhoA-specific cleavage sites in fragments F1 (Tyr-Leu-Glu-Val↓Lys-Gly-Thr-Gly) and F4 (Thr-Lys-Leu-Met↓Thr-Arg-Leu-Thr) are not in the Merops peptidase database, indicating that HhoA belongs to a subfamily of the chymotrypsin family of peptidases that has its own unique substrate specificity. The identified cleavage sites are located near to the N-terminus of the protein (Figure 4B) and large fragments (32 to 24 kDa) remain after rHhoA action. Currently, we cannot exclude the need for additional factors/proteases to enhance or continue with the redox-dependent degradation of PsbO. However, addition of different combinations of recombinant Deg proteases (rHtrA or rHhoB with rHhoA) to reduced PsbO did not lead to a faster or further degradation of the protein in spinach (Figure S4).

**Figure 5:**
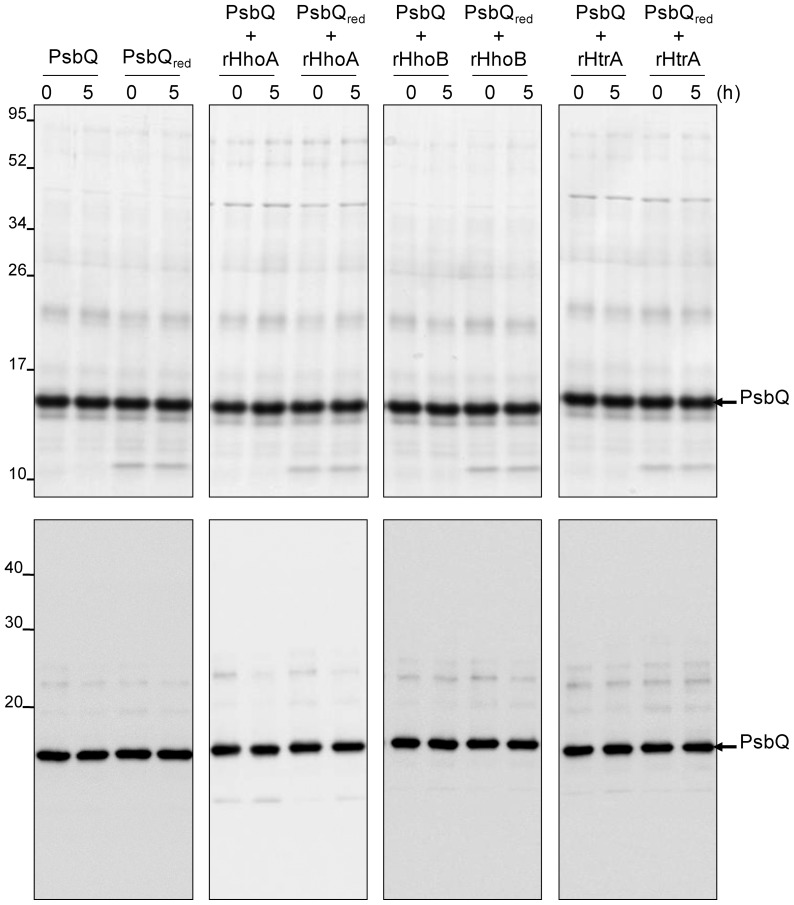
PsbQ is not degraded by the recombinant Deg proteases. PsbQ was isolated from spinach and incubated for 5 h in the absence or the presence (PsbQ_red_) of the thioredoxin system, either with no protease (panel 1) or with rHhoA (panel 2), rHhoB (panel 3), or rHtrA (panel 4). After SDS-PAGE, the gel was either stained with CBB (upper panels, 6 µg of protein loaded) or blotted and immunostained using an antibody directed against PsbQ (lower panels, 1.5 µg of protein loaded).

To test the substrate specificity of the redox-dependent degradation performed by rHhoA, another extrinsic protein of the manganese-stabilizing complex of PSII, the PsbQ protein was isolated from PSII membrane fragments and incubated for 5 h at 37°C in the presence or absence of the thioredoxin system (denoted PsbQ_red_ and PsbQ, respectively). As shown in Figure 5, no degradation was observed, either on the CBB-stained gel (upper panels) or in the immunoblot using antibodies directed against PsbQ (lower panels); the additional band with molecular mass of 12 kDa in the PsbQ_red_ fractions corresponded to thioredoxin. Addition of recombinant HhoA, HhoB, or HtrA did not result in any degradation of PsbQ, neither in the presence nor in the absence of the thioredoxin system (panels 2–4). As spinach PsbQ lacks any cysteine residue, a thiol-dependent conformational change that facilitates degradation of this protein would not be expected, and the absence of any redox-effect in the control assay using PsbQ supports our hypothesis that the activities of rHhoA, rHhoB, and rHtrA are not regulated by thioredoxin.

### PsbO bound to PSII is protected against degradation in reducing conditions

**Figure 6:**
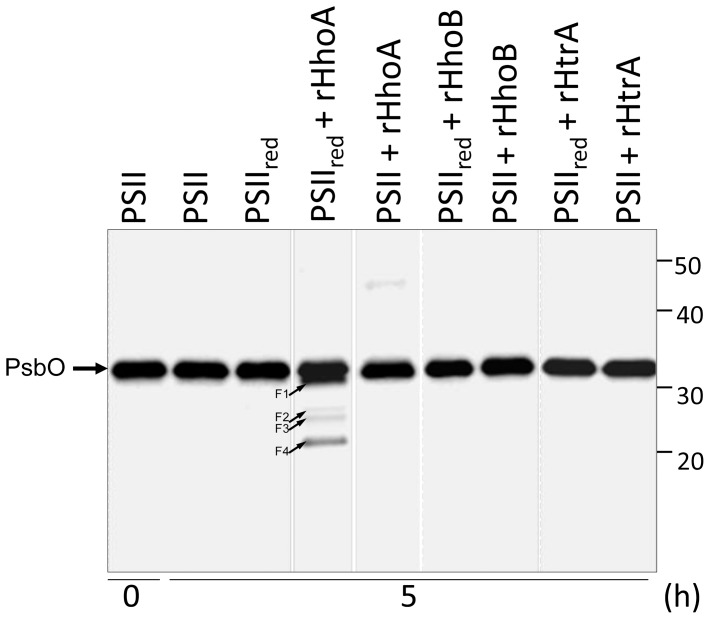
PsbO bound to PSII is protected against degradation by native proteases. PSII membrane fragments from spinach were isolated and incubated for 5 h in the absence (PSII) or the presence (PSII_red_) of reduced thioredoxin, either with no protease or with rHhoA, rHhoB, or rHtrA. After SDS-PAGE, the proteins were blotted and immunostained using an antibody directed against PsbO. Small arrows indicate PsbO degradation fragments (F1 to F4).

PsbO is assembled into PSII. However, a pool of free PsbO has been shown to exist in the thylakoid lumen [43]. Under reducing conditions, unassembled PsbO was degraded without addition of recombinant proteases (Figure 3A), which is consistent with the presence of Deg proteases in the thylakoid lumen. To investigate if PsbO attached to PSII can also be degraded, PSII membrane fragments were isolated from spinach leaves and incubated for 5 h in the presence or absence of reduced thioredoxin. After separation of the proteins by SDS-PAGE, PsbO was immunostained (Figure 6). No degradation of PsbO was observed in the PSII fraction of spinach; neither in the absence nor in the presence of reduced thioredoxin (Figure 6). Addition of recombinant rHhoB or rHtrA to non-reduced or reduced PSII did not lead to any degradation. However, addition of rHhoA resulted in slight, redox-dependent degradation of PsbO; evidenced by the appearance of the previously described fragments after 5 h of incubation (Figure 6). However, this degradation was slower and weaker than for isolated PsbO (Figure 4A), suggesting that the protein is protected against degradation when it is bound to PSII. We believe that the PSII preparation contained some unassembled PsbO that served as a substrate for rHhoA.

## Discussion

### Degradation of PsbO is thioredoxin-dependent

The PsbO protein of plants [29] and cyanobacteria [63] is a protein of elongated shape containing two major domains: The very stable domain I is composed of eight antiparallel β strands which form a cylinder with hydrophobic amino acid residues in its central part. Domain II mainly consists of random coils and turns and a distinct α helix. The hydrophilic loops are stabilized by interaction with other PSII proteins and are flexible in the non-assembled protein. PsbO contains two conserved cysteine residues, Cys28 and Cys51 in spinach [38], that form an S-S bridge between the N-terminal loop and the β1 strand. While the function of these two cysteins is controversial, it was recently found that they are redox sensitive and might be relevant for the function of PsbO [20], [46]–[48].

The PsbO protein has been found to be remarkably stable when exposed to different temperatures and pHs [44], [64]. However, in Arabidopsis both PsbO proteins–PsbO1 and PsbO2–become unstable after reduction by thioredoxin, and are degraded [20]. In this study, we have confirmed the previous results for Arabidopsis and shown that also the PsbO of spinach and of the cyanobacterium *Synechocystis* 6803 are redox-dependently degraded. The relevance of these observations for the turnover of PsbO under physiological conditions is supported by the recent finding that PsbO levels in the *lto1* mutant of Arabidopsis are clearly lower than in wild-type plants [65]. The *lto1* gene encodes for a lumen thiol oxidoreductase (LTO1) that catalyzes disulfide formation of thylakoid proteins on the lumen side of the thylakoid membrane. In the *lto1* mutant, oxidation of reduced PsbO is inhibited, which would facilitate degradation of this protein and explain the lower levels of PsbO in this mutant than in the wild type [65].

Recombinant HhoA from *Synechocystis* 6803 cleaves PsbO from spinach in the N-terminal region specifically before (F1) and after (F4) the disulfide bridge, which is consistent with the redox-dependence of this process. This degradation is very specific, and other extrinsic PSII proteins such as PsbQ are not degraded. The cleavage sites of the HhoA-specific fragments F1 (YLEV-KGTG) and F4 (TKLM-TRLT) are new and not in the Merops peptidase database. This suggests that HhoA belongs to a subfamily of the chymotrypsin family of peptidases that have their own unique substrate specificity.

### Degradation kinetics

In purified chloroplast lumen of Arabidopsis, degradation of PsbO has been shown to be complete within 5 h of the onset of reduction by thioredoxin [20]. Similar results were obtained for PsbO from spinach, but the rate of the degradation was slower than for the PsbO proteins from Arabidopsis. This might be due to a more negative redox potential of PsbO in spinach than in Arabidopsis. In addition, the native protease activity responsible for PsbO degradation might need additional factors/proteases to enhance the redox-dependent degradation.

### PsbO degradation by Deg proteases

Various chloroplast-located Deg proteases are known to be involved in the degradation of photosynthetic proteins under stress conditions, and the main target seems to be the reaction-center protein D1 [13]–[17]. Recombinant Arabidopsis Deg1 was also to some extent able to degrade recombinant PsbO [18]. These results appear to be contradictory to ours, as unreduced PsbO was degraded by Deg1. However, recombinant PsbO is, at least partially, produced in its reduced form. We observed degradation of recombinant PsbO from spinach and *Synechocystis* 6803 without addition of the thioredoxin system (not shown). However, a control using thiol labeling with monobromobimane showed that recombinant PsbO from *Synechocystis* 6803 mainly contained reduced cysteine thiols (Figure S1), which indicated that *E. coli* is not able to correctly form the disulfide bridge of PsbO. Consistent with these observations, a recent study in Arabidopsis showed that Deg1 is not responsible for degradation of mature PsbO *in vivo*, but acts downstream by degrading its fragments [66].

### Is PsbO degraded in its free form or bound to PSII?

The conformations of PsbO in solution differ from those when it is bound to PSII [42], [67], [68]. Conformational changes of PsbO under different light conditions and heat treatments affect its binding ability [37], which might trigger its degradation. It has been shown that the disulfide bridge is important for folding and rebinding of PsbO to PSII [40]; and binding of PsbO to PSII seems to protect it from degradation in spinach (Figure 6). In PSII isolated from *Synechocystis* 6803, a moderate degradation of PsbO was visible, which was probably due to co-isolated HhoA or other proteases (Figure 1B and 1C). We believe that by degrading free PsbO present in the PSII fractions, the equilibrium between bound and free PsbO is changed, so that more PsbO dissociates from PSII and therefore more PsbO can be degraded by the (recombinant or native) Deg proteases. Free, unassembled PsbO appears to be destabilized after reduction of its disulfide bridge and becomes accessible to proteolysis. While rHhoA was able to degrade PsbO from spinach in a redox-dependent manner, it is still unclear what roles the lumenal Deg proteases Deg1, Deg5, and Deg 8 play in the degradation of PsbO. However, Deg1 does not appear to be involved in the primary cleavage of PsbO [66].

For PsbO from *Synechocystis* 6803, Figure 1B shows that PsbO in PSII-enriched membrane fragments is essentially stable, but becomes degraded under reducing conditions. The presence of externally added recombinant HhoA clearly enhances the degradation of PsbO under reducing conditions, while for externally added rHhoB and rHtrA no effect was observed. These observations suggest that PsbO of *Synechocystis* 6803 is a substrate for rHhoA, but not for rHhoB and rHtrA.

### Subcellular location of the Deg proteases in *Synechocystis* 6803

The location of the three Deg proteases in *Synechocystis* 6803 has not previously been determined. In contrast to green plants, protein sorting in cyanobacteria is poorly understood, and reliable methods for separating the periplasm and the thylakoid lumen do not yet exist [69]. Our immunological studies have identified all three proteases attached to the plasma membrane, although HhoA and HtrA also seem to be associated with the thylakoid membrane (Figure 2B). It has been shown previously that all three Deg proteases have overlapping functions [25], which is supported by a common sub-localization within the cell. Components of the reaction centres of PSI and PSII as well as PsbO have previously been identified in the plasma membrane of *Synechocystis* 6803 [53], [70]. Additionally, convergence sites between the thylakoid membranes and the plasma membrane have been observed [71], which allow the Deg proteases located at the plasma membrane access to PSII *in vivo*. A co-localization of the cyanobacterial Deg proteases within the plasma membrane and the thylakoid membrane (partly close to PSII) is therefore possible, although a localization within PSII is not likely, as the levels of the Deg proteases in the PSII-enriched fraction were very low. In Arabidopsis however, Deg1 has been co-isolated with PSII and has been shown to interact specifically with the reaction-centre protein D2 [17].

In summary, it had been proposed that the cyanobacterial Deg proteases were involved in response to heat- and light-stress [22], [24], [25], [72], but physiological substrates of cyanobacterial Deg/HtrA proteases had not been identified. Here, we were able to show biochemically that PsbO from spinach and from *Synechocystis* 6803 could be substrates for the cyanobacterial Deg protease HhoA, and that the degradation of PsbO by rHhoA is likely redox-dependent. Further research is needed to determine whether redox-dependent proteolysis of PsbO by Deg proteases plays a role in PSII assembly and repair. Genetic evidence supporting our finding is currently lacking. Our efforts to investigate the possible accumulation of PsbO in a triple knockout mutant (hhoA-, hhoB- and htrA-) in *Synechocystis* both under high light or high temperature stress were not successful. Further experiments in a suitable homologous system in plants will be needed to address this question.

## Materials and Methods

### Lumen, PSII, PsbO, and PsbQ purification from spinach and leaves

Spinach leaves were bought on the local market. Arabidopsis plants were grown as described previously [20]. Thylakoid membranes and PSII membrane fragments were isolated from spinach leaves essentially as described previously [73], [74]. Chloroplast lumen from spinach and Arabidopsis leaves was prepared as described previously [62]. PsbO was released from the PSII membrane fragments by salt-wash treatment after removal of PsbQ and PsbP [75]. PsbQ was purified from this fraction, and obtained in 1 *M* NaCl, 10 m*M* MES, pH 6.5. The buffer was switched to 50 m*M* Tris-HCl, pH 7.5 and loaded onto a Q-Sepharose column equilibrated in the same buffer. PsbQ was recovered in the flow through; the buffer was switched to 50 m*M* CAPS, pH 10 and loaded onto a second Q-Sepharose column equilibrated in the same buffer. After washing with five bed volumes of starting buffer, the proteins were eluted with 600 m*M* NaCl, 50 m*M* CAPS, pH 10. The PsbO fraction obtained in 0.8 *M* Tris-HCl buffer, pH 8.4 was diluted with Milli-Q water to 50 m*M* Tris-HCl and concentrated by binding to a small Q-Sepharose column (0.5×1 cm) equilibrated with 50 m*M* Tris-HCl buffer, pH 8.4. Bound proteins were eluted with 600 m*M* NaCl in the same buffer. Fractions were analyzed by SDS-PAGE [76], and those containing PsbQ or PsbO were pooled before concentration and desalting by ultrafiltration with 3-kDa and 10-kDa cutoffs, respectively (Amicon Ultra-15, Millipore).

### Membrane and PSII purification from *Synechocystis* 6803 cells

Soluble and membrane fractions of *Synechocystis* 6803 were separated by a combination of sucrose-density centrifugation and aqueous two-phase partitioning [56]. PSII containing His-tagged CP47 was isolated from the HT3 strain of *Synechocystis* 6803 [51]. Cells were grown in liquid BG-11 medium in the presence of 25 µg ml^−1^ kanamycin under 50 µmol photons m^−2^ s^−1^, with air bubbling at 30°C. His-tagged PSII was purified as described previously [52] except that imidazole was used instead of histidine to elute the proteins bound to the Ni-NTA column. After two washing steps with 15 and 35 m*M* imidazole in buffer A [50 m*M* MES-NaOH, pH 6.0; 10 m*M* MgCl_2_; 5 m*M* CaCl_2_; and 25% (v/v) glycerol] with 0.04% (w/v) dodecyl maltoside, proteins were eluted with 100 m*M* imidazole in buffer A with 0.04% (w/v) dodecyl maltoside. The eluted fractions were pooled and the sample was concentrated and equilibrated with buffer A by ultrafiltration (50-kDa cutoff, Amicon Ultra-15, Millipore).

### Overexpression and purification of recombinant Deg proteases from *Synechocystis* 6803

The construct overexpressing *Synechocystis* rHhoA protease and lacking the predicted signal peptide at the N-terminal end was developed by Huesgen et al. [72]. Cloning and purification of recombinant HtrA and HhoB proteases followed a similar strategy [50]. Transformed *E. coli* cells were grown in LB liquid medium containing 100 µg ml^−1^ ampicillin at 19°C for 10 h, and expression of recombinant proteases was induced by the addition of 0.1 m*M* isopropyl-1-thio-D-galactoside (IPTG). Cultures were further grown overnight at 19°C, then harvested by centrifugation (5 000 *g* for 10 min at 4°C). Cell pellets were resuspended in 10 to 20 ml of binding buffer (50 m*M* HEPES-NaOH, pH 8.0 and 300 m*M* NaCl) and stored at −20°C. Cells were lyzed on ice by 10 to 15 repeats of 10-s sonication with 20-s cooling interval and centrifuged (26 000 *g*, 1 h, 4°C). Recombinant proteases were purified at 5°C from filtered soluble cell lysates (0.2-μm filters) using His GraviTrap affinity columns (GE Healthcare) according to manufacturer's instructions. Application in binding buffer was followed by a washing step with 10 ml of binding buffer containing 60 m*M* imidazole and an elution step with 5 ml of elution buffer (50 m*M* HEPES-NaOH, pH 8.0; 300 m*M* NaCl; and 500 m*M* imidazole). Protein concentrations were determined by Bradford protein assay (Biorad) with BSA solutions as standard.

### Proteolytic activity of recombinant Deg proteases

Purified recombinant proteases were incubated with an excess of β-casein in 250 m*M* buffer Tris-HCl, pH 7.0, supplemented with 20 m*M* CaCl_2_ for 30 min at 40°C. After reaction, proteins were analyzed by SDS-PAGE, as described below.

### Overexpression and purification of recombinant PsbO from Synechocystis 6803

Specific primers (5′ CGCGCGGCAGCCATATGGTTGATAAGAGCCAGCTTAC 3′and 5′ GGATCCTCGAGCATATGTTAAACATCGGTGTCCAC 3′) were designed to amplify the *psbO* gene (*sll0427*) lacking the signal peptide from genomic DNA isolated from *Synechocystis* 6803. The PCR product was cloned into a pET-15b vector (Novagen) to produce a fusion protein containing an N-terminal His-tag using the In-Fusion Advantage PCR cloning kit (Clontech), following the manufacturer's instructions. Chemo-competent cells of the expression strains Rosetta 2 (DE3) or Rosetta-Gami 2 (DE3) (Novagen) were transformed with the plasmid and selected on LB agar containing 50 µg ml^−1^ carbenicillin and 34 µg ml^−1^ chloramphenicol [for Rosetta 2 (DE3) cells] or 50 µg ml^−1^ carbenicillin, 34 µg ml^−1^ chloramphenicol, 12.5 µg ml^−1^ tetracyclin, and 50 µg ml^−1^ streptomycin [for Rosetta-Gami 2 (DE3) cells]. 2–l cultures of selected colonies from each strain [7S from Rosetta 2 (DE3) cells and 2RG from Rosetta-Gami 2 (DE3) cells] were grown in LB supplemented with the corresponding antibiotics, at 30°C until OD600 = 0.5–0.6, at which point expression was induced by addition of 1 m*M* IPTG. Five hours after induction, cells were harvested by centrifugation at 10 000 *g* for 10 min at 4°C and resuspended in 30 ml of 20 m*M* sodium phosphate buffer, pH 8.0 containing 300 m*M* NaCl (buffer B). For the purification of the His-tagged PsbO, cells were broken on ice by 10 cycles of 10-s sonication with 20-s intervals of cooling, and centrifuged at 26 000 *g*, 1 h, 4°C. Supernatant was filtered through 0.2-µm filters and loaded onto a His GraviTrap affinity column (GE Healthcare) equilibrated in buffer B. After washing with 10 column volumes of buffer B containing 60 m*M* imidazole, bound proteins were eluted in 1-ml fractions with 10 column volumes of 500 m*M* imidazole prepared in buffer B. Fractions were analyzed by SDS-PAGE and those containing the PsbO peak were pooled. The sample was concentrated and the buffer switched to 50 m*M* Tris-HCl, pH 7.5 by ultrafiltration (10-kDa cutoff, Amicon Ultra-15, Millipore).

### PsbO and PsbQ degradation assay

The activity of *Synechocystis* rHhoA, rHhoB, and rHtrA proteases against reduced PsbO or PsbQ was assayed *in vitro* by incubating different samples isolated from spinach leaves (150 µg of purified PsbO or 100 µg of purified PsbQ) or from *Synechocystis* 6803 (125 µg of protein of PSII-enriched fraction isolated from the HT3 strain).

The reaction mixture, with a final volume of 250 µl, contained 50 m*M* Tris-HCl buffer, pH 7.5; 4 µg of *E. coli* thioredoxin (Sigma); 3.5 µg of *E. coli* thioredoxin reductase (Sigma); and 1.6 m*M* β-NADPH (Sigma) [77]. After a pre-incubation period of 15 min at 37°C, 3 µg (1×; 1∶50 enzyme to substrate ratio) or 15 µg (5×; 1∶10) of purified recombinant HhoA, HhoB, or HtrA protease were added (0 h) and incubation proceeded at the same temperature. Samples of 45 µl were taken at 0 and 5 h, mixed with 15 µl of 4× sample buffer and heated at 95°C for 5 min.

PsbO degradation in isolated PSII membrane fragments of spinach (400 µg Chl) was assessed in the absence or the presence of the reducing system as described above in a final volume of 200 µl. For the lumen fractions of spinach and Arabidopsis, the assay was performed as described in Hall et al. [20].

### Polyacrylamide gel electrophoresis and immunoblotting

Proteins were separated on 14% SDS-PAGE [76] and visualized by CBB staining. For immunoblotting, electrophoretically separated proteins were transferred to PVDF membranes [78]. After electroblotting, the membranes were blocked using 10% (w/v) milk in PBS and incubated overnight at 4°C with a dilution of 1∶10 000 (*Synechocystis* 6803 samples) or 1∶20 000 (spinach samples) of primary anti-PsbO antibody (Agrisera, Sweden) or 1∶5 000 of anti-PsbQ antibody (Agrisera, Sweden). For detection of Deg proteases, a 1∶10 000 dilution of polyclonal antisera prepared in rabbits (Agrisera, Sweden) against recombinant HhoA, HhoB, or HtrA, purified as described above, was used for 2 h at room temperature. Antibodies directed against CP47 and NrtA were used as described previously [70]. In all cases, the membranes were probed with a 1∶150 000 dilution of goat anti-rabbit secondary antibody conjugated to horseradish peroxidase (Biorad) and developed using the ECL Advance Western blotting reagents from GE Healthcare.

### Mass spectrometric analysis of PsbO

In the CBB-stained SDS-PAGE of the lumen fraction of spinach, the identity of the band corresponding to PsbO was confirmed using mass spectrometry according to Yao et al. [79] and Jun et al. [80].

### N-terminal sequence analysis of the spinach PsbO fragments

N-terminal sequence analysis of the spinach PsbO fragments produced by rHhoA was carried out by the Edman degradation method at the Protein Analysis Center (PAC) (Karolinska Institute, Stockholm, Sweden). A sample of 50 µg of purified PsbO was digested with 2 µg of rHhoA in the presence of the reduced *E. coli* thioredoxin, as described above, for 5 h at 37°C. After SDS-PAGE, the proteins were transferred to a PVDF membrane, stained with 0.1% (w/v) CBB in 50% (v/v) methanol and the bands corresponding to the fragments were analyzed [81].

### Monobromobimane labeling of sulfhydryl groups in spinach lumenal proteins and recombinant PsbO from *Synechocystis*

Monobromobimane (mBBr) labeling of sulfhydryl groups in spinach lumenal proteins and recombinant PsbO from *Synechocystis* (isolated from strains 7S or 2RG) was performed essentially as described previously [77]. A sample of 10 µg of protein was incubated with 5 µl of 20 m*M* mBBr prepared in acetonitrile, in 50 m*M* buffer Tris-HCl, pH 8, with a final volume of 55 µl. The labeling reaction was conducted at room temperature for 15 min in darkness. To stop the reaction, 10 µl of 100 m*M* β-mercaptoethanol was added to the samples. Proteins were precipitated with five volumes of 100% acetone at −20°C for 2 h and recovered by centrifugation at 16 000 *g* for 15 min. The pellet was washed with acetone, air dried, solubilized in sample buffer, and heated at 80°C for 10 min. After SDS-PAGE, the fluorescence of the labeled proteins was visualized by UV, and the gels were stained with CBB. To attempt the total reduction of the disulfide bonds, PsbO samples were pre-incubated in the presence of 2.5 m*M* dithiotreitol (DTT) for 15 min at room temperature prior to mBBr treatment. To oxidize all the SH groups, samples of 6 m*M* recombinant PsbO were incubated in the presence of 35 m*M* CuSO_4_ for 2 h at room temperature and dialyzed against two changes of 50 m*M* Tris-HCl, pH 8, prior to mBBr treatment.

### Quantification of gels and blots signals

The integrated density values of the bands was measured using Image J software, free available at http://rsb.info.nih.gov/ij/index.html.

## Supporting Information

Figure S1 **Recombinant PsbO is reduced even in the absence of reducing agents.** Recombinant PsbO of *Synechocystis* 6803 was isolated from the strains 7S or 2RG and incubated with monobromobiname (mBBr) to label the sulfhydryl groups. To reduce or oxidize PsbO, samples were pre-treated with 2.5 mM DTT or 35 mM CuSO_4_, respectively, previous to mBBr labeling. Ten micrograms of protein were loaded per lane. After SDS-PAGE proteins were visualized by UV (upper panel) and stained with CBB (lower panel).
(TIF)

Figure S2 **Alignment of PsbO sequences.** Multiple sequence alignment of PsbO1 (ARAB 1, accession number AED98230.1) and PsbO2 (ARAB 2, accession number AEE78714.1) from *A. thaliana*, and PsbO from spinach (accession number P12359.1) and *Synechocystis* 6803 (SYNECHO, accession number NP441796.1). The alignment was generated using ClustalW2 software. Asterisk indicates fully conserved residues, colon and period indicate strong and weak conservation, respectively, as defined by ClustalW2.
(TIF)

Figure S3 **Time course degradation of PsbO in the presence of recombinant Deg proteases from** ***Synechocystis*** **sp. PCC 6803.** PsbO was isolated from spinach leaves and incubated in the absence (PsbO) or the presence (PsbO_red_) of the complete thioredoxin system for 10 h without addition of recombinant proteases (A) or after addition of rHhoA (B), rHhoB (C) or rHtrA (D). Proteases were used at the standard enzyme to substrate ratio of 1∶50 or at a ratio five times higher (1∶10) referred as 5X. After SDS-PAGE the proteins (9 µg) were stained with CBB, the integrated density of the PsbO band was quantified using Image J software and represented as the residual amount taking the 0 h band as 100%.
(TIF)

Figure S4 **Redox-dependent degradation of PsbO in the presence of combinations of recombinant Deg proteases from** ***Synechocystis*** **sp. PCC 6803.** PsbO was isolated from spinach leaves and incubated in the absence or the presence (PsbO_red_) of the complete thioredoxin system together with rHhoA and rHhoB, rHhoA and rHtrA or rHtrA and rHhoB for 5 h. Arrowheads indicate PsbO degradation fragments (1 to 4) as described in the text.
(TIF)
