# Supplementary figures and images for: Degradation of PsbO by the Deg Protease HhoA Is Thioredoxin Dependent
Source: PLoS One. 2012 Sep 19;7(9):e45713. doi: 10.1371/journal.pone.0045713 (PMC3446894; doi:10.1371/journal.pone.0045713)

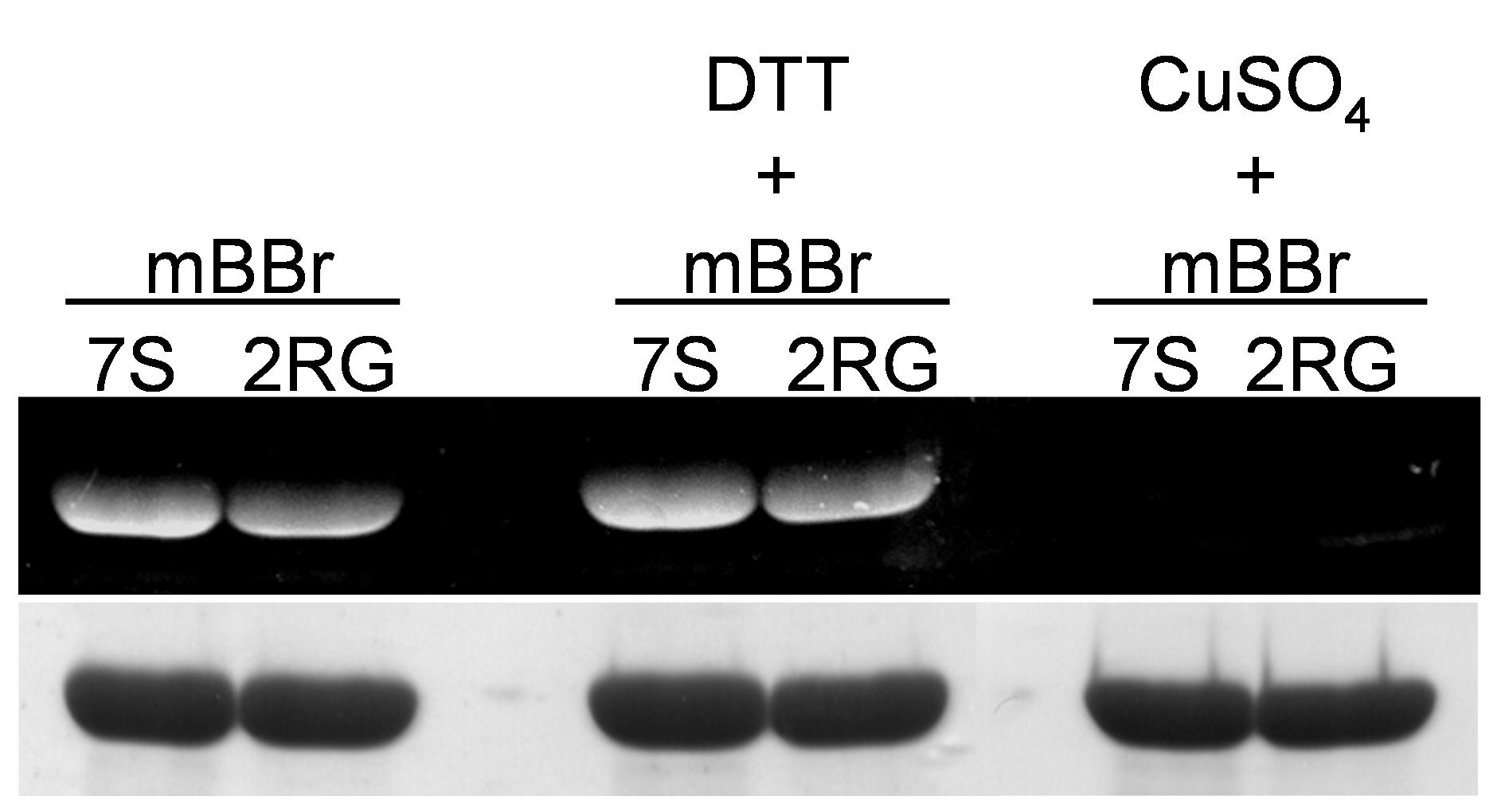

Supplement: Figure S1 — Recombinant PsbO is reduced even in the absence of reducing agents. Recombinant PsbO of Synechocystis 6803 was isolated from the strains 7S or 2RG and incubated with monobromobiname (mBBr) to label the sulfhydryl groups. To reduce or oxidize PsbO, samples were pre-treated with 2.5 mM DTT or 35 mM CuSO4, respectively, previous to mBBr labeling. Ten micrograms of protein were loaded per lane. After SDS-PAGE proteins were visualized by UV (upper panel) and stained with CBB (lower panel). (TIF) [file pone.0045713.s001.tif]

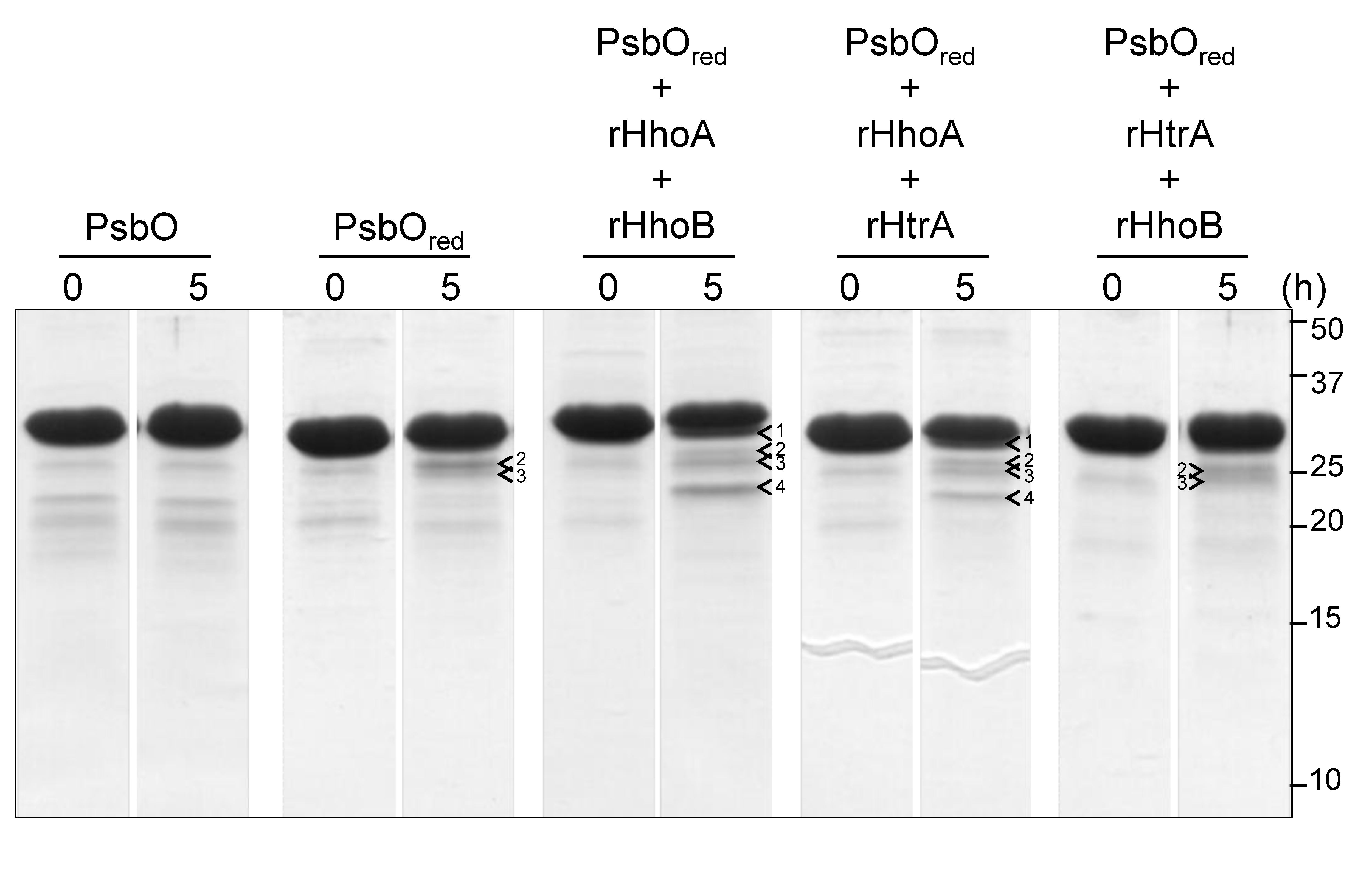

Supplement: Figure S4 — Redox-dependent degradation of PsbO in the presence of combinations of recombinant Deg proteases from Synechocystis sp. PCC 6803. PsbO was isolated from spinach leaves and incubated in the absence or the presence (PsbOred) of the complete thioredoxin system together with rHhoA and rHhoB, rHhoA and rHtrA or rHtrA and rHhoB for 5 h. Arrowheads indicate PsbO degradation fragments (1 to 4) as described in the text. (TIF) [file pone.0045713.s004.tif]
